# Transcription profiling of butanol producer Clostridium beijerinckii NRRL B-598 using RNA-Seq

**DOI:** 10.1186/s12864-018-4805-8

**Published:** 2018-05-30

**Authors:** Karel Sedlar, Pavlina Koscova, Maryna Vasylkivska, Barbora Branska, Jan Kolek, Kristyna Kupkova, Petra Patakova, Ivo Provaznik

**Affiliations:** 1 Department of Biomedical Engineering, Brno University of Technology, Technicka 12, 616 00 Brno, Czechia; 2 Department of Biotechnology, University of Chemistry and Technology Prague, Technicka 5, 166 28 Prague, Czechia; 3 Institute of Aquaculture and Protection of Waters, University of South Bohemia in České Budějovice, Na Sádkách 1780, 370 05 České Budějovice, Czechia; 4 Department of Biochemistry and Molecular Genetics, University of Virginia Health System, Charlottesville, VA 22908 USA

**Keywords:** *Clostridium beijerinckii* NRRL B-598, RNA-Seq transcriptome, ABE fermentation

## Abstract

**Background:**

Thinning supplies of natural resources increase attention to sustainable microbial production of bio-based fuels. The strain *Clostridium beijerinckii* NRRL B-598 is a relatively well-described butanol producer regarding its genotype and phenotype under various conditions. However, a link between these two levels, lying in the description of the gene regulation mechanisms, is missing for this strain, due to the lack of transcriptomic data.

**Results:**

In this paper, we present a transcription profile of the strain over the whole fermentation using an RNA-Seq dataset covering six time-points with the current highest dynamic range among solventogenic clostridia. We investigated the accuracy of the genome sequence and particular genome elements, including pseudogenes and prophages. While some pseudogenes were highly expressed, all three identified prophages remained silent. Furthermore, we identified major changes in the transcriptional activity of genes using differential expression analysis between adjacent time-points. We identified functional groups of these significantly regulated genes and together with fermentation and cultivation kinetics captured using liquid chromatography and flow cytometry, we identified basic changes in the metabolism of the strain during fermentation. Interestingly, *C. beijerinckii* NRRL B-598 demonstrated different behavior in comparison with the closely related strain *C. beijerinckii* NCIMB 8052 in the latter phases of cultivation.

**Conclusions:**

We provided a complex analysis of the *C. beijerinckii* NRRL B-598 fermentation profile using several technologies, including RNA-Seq. We described the changes in the global metabolism of the strain and confirmed the uniqueness of its behavior. The whole experiment demonstrated a good reproducibility. Therefore, we will be able to repeat the experiment under selected conditions in order to investigate particular metabolic changes and signaling pathways suitable for following targeted engineering.

**Electronic supplementary material:**

The online version of this article (10.1186/s12864-018-4805-8) contains supplementary material, which is available to authorized users.

## Background

While a less costly petroleum refinery still represents the main source of fuels and chemicals, limited natural resources and nature protection have increased attention to sustainable production of bio-based products. These trends make biorefinery the future lucrative producer of renewable fuels and chemicals. Especially, the microbial production of solvents such as acetone, butanol, and ethanol (ABE) is currently of great interest [1]. Solventogenic *Clostridia* are widely studied for their ability to produce biofuels from biomass in ABE fermentation [2]. Unfortunately, different genera or even strains of these rod-shaped, gram-positive anaerobes show substantial differences in phenotypic traits, i.e. the ability to utilize different substrates and to produce different substances. Thus, the findings acquired using model organisms such as *C. acetobutylicum* ATCC 824 [3], *C. pasteurianum* DSM 525 [4], or *C. beijerinckii* NCIMB 8052 [5] cannot be applied in general. Fortunately, thanks to a massive reduction in sequencing costs, a wide range of complete or at least draft genomes of solventogenic *Clostridia* are now available. These include various strains of *C. acetobutylicum*, *C. aurantibutyricum*, *C. beijerinckii*, *C. diolis*, *C. felsineum*, *C. pasteurianum*, *C. puniceum*, *C. roseum*, *C. saccharobutylicum*, and *C. saccharoperbutylacetonicum* [6]. *C. beijerinckii* strains, utilizing a wider range of substrates for solvent production seem to be the most robust, i.e. able to endure a wide range of environmental conditions, among these [7].

However, the knowledge of the genomic sequence itself does not provide any information regarding the gene regulation, which is crucial to improvements of the strains for industrial application. The study of gene expression is therefore irreplaceable in genome engineering. Current whole transcriptome sequencing technology, referred to as RNA-Seq, allows the study of transcription on a genome-wide scale with an unlimited dynamic range, compared to the older microarrays, which only enabled researchers to track preselected genes [8]. In this paper, we present transcriptome dynamics during the cultivation of the promising butanol producer, *C. beijerinckii* NRRL B-598 [9] (formerly misidentified as *C. pasteurianum* NRRL B-598 [10]) as a result of RNA-Seq profiling. Until now, only the transcription of six selected genes involved in sporulation and solvent production was studied for this strain using RT-qPCR, yet the study supported the theory that solventogenesis is not regulated in the same way in all solventogenic clostridia [11]. Here, we further investigate the specifics of the strain *C. beijerinckii* NRRL B-598. The obtained transcriptome data includes the whole life cycle of the strain and therefore covers changes in metabolism, i.e. acidogenesis, solventogenesis and their transition state. Together with the sporulation cycle and other significant events such as changing motility and adaptation to acid/solvent stress, the whole fermentation process is reflected in this dataset. Flow cytometry, combined with fluorescent staining [12], has enabled insights into population heterogeneity and HPLC analysis of metabolites/substrate; plus, growth curve data has allowed us to better interpret the biological meaning. Moreover, the RNA-Seq technology has allowed us to study not only the temporal transcription of any gene but also to explore the accuracy of the current genome annotation. Compared to the transcription profiling of the strain *C. beijerinckii* NCIMB 8052, we reached a dynamic range that was approximately 10 times higher. To increase the robustness and validity of the experiment, each of the time-points was represented by three biological replicates rather, than verification using qPCR [13].

## Results

### Cultivation and fermentation kinetics

**Fig. 1:**
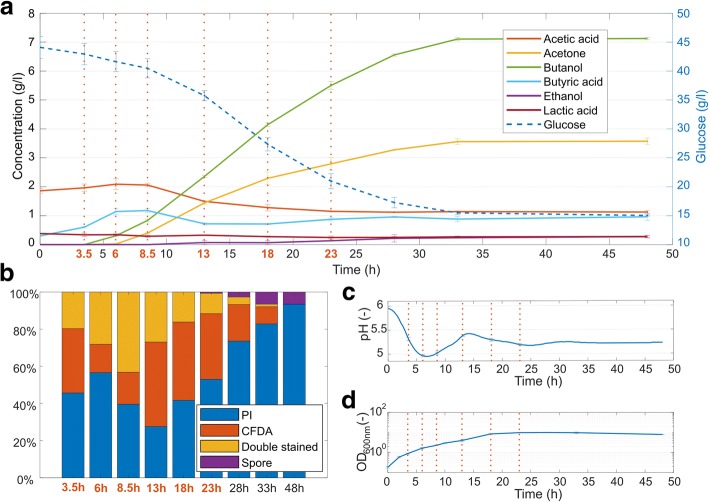
Cultivation and fermentation characteristics of *Clostridium beijerinckii* NRRL B-598. (**a**) The concentration of glucose, solvents and acids during ABE fermentation. (**b**) Flow cytometry – the distribution of cells within the population according to their fluorescence pattern for combined staining using PI and CFDA. (**c**) pH curve for respective cultivation. (**d**) Cell growth measured as optical density at 600 nm. Values represent the mean of the biological replicates and error bars represent the standard deviations. Time-points (T1–T6) for samples subjected to RNA expression analysis are indicated by red vertical dotted lines and/or by red text labels

The fermentation profile of *C. beijerinckii* NRRL B-598 showed a typical two-stage course of metabolites formation with acid production in the first period followed by solvents formation (see Fig. 1a). Six time-points (T1–T6) were selected for RNA-Seq analysis to cover all metabolic stages within a period of 23 h. The latter stages were not analyzed due to a high percentage of dead and lysing cells (Fig. 1b) causing an insufficient quality of RNA samples for RNA-Seq. Individual sampling points were selected based on the fermentation pattern, which was monitored on-line as changes in a pH course (Fig. 1c). The first sample was collected after an approximate five-fold increase in optical cell density (Fig. 1d) while a sharp decrease in pH occurred, so only acidogenic, non-sporulating and mostly motile cells were expected to be present in the sample. The second time-point was proposed to cover a transient physiological state between acidogenesis and solventogenesis, which was indicated by a pH breakpoint and corresponded to the highest concentration of acids in media along with the onset of solvent formation. No cell-thickening or pre-spore formation was observed at this stage. The third sample set was withdrawn during the period of the most progressive rise in pH, suggesting a high rate of reutilization of the acids, together with solvent formation. Granulose accumulation and early phases of sporulation were observed at this stage (see Additional file 1). The second pH breakpoint was covered by the fourth sample, where the rise in pH ceased and pH again started to decline, indicating a change in metabolism. However, there was no apparent increase in the production of acids in the fermentation data. The remaining two samples were taken at the regular time-intervals, in order to cover all stages of ABE fermentation as well as the sporulation cycle. Overall culture fitness and spore formation was monitored by flow cytometry (FC) and the combined staining of cell culture by membrane disruption and enzyme activity indicators: propidium iodide (PI) and carboxyfluorescein diacetate (CFDA), respectively. A relatively high amount of double-stained cells was present in the culture at all stages. A previous study by Kolek et al. [12] considered these double-stained cells as an active population consisting of cell doublets and sporulating cells; therefore, only PI-positive cells were counted as dead cells. The staining pattern of the Clostridium culture at different time-points revealed dynamic changes in proportion of active cells within the first 13 h, with a detectable drop at the period with the lowest pH (the sixth hour), thus supporting the presumption that cells are highly-stressed by the presence of organic acids together with a low pH (when values slightly below pH 5 were reached). After the 13th hour, viability gradually decreased and during the 23rd hour the first mature spores, released from mother cells, were observed. The FC data provided a better insight into viability changes compared to sole OD measurements, according to which the culture kept on growing steadily until the 18th hour. The only noticeable changes in the OD measurements are the two slowdowns during the acidogenesis/solventogenesis transient states. The FC data clearly shows that culture viability had already started to decline at around the 13th hour, which corresponds to the apparent decrease in the number of regulated genes from that time.

A proportion of viable cells determined by FC was used to calculate the specific glucose consumption rate relating only to the active portion of clostridium culture (see Table 1). The amount of glucose consumed per time and biomass unit could help to elucidate the differences in expressions of glycolysis-related genes. The highest number of 5.16 g of utilized glucose per gram of active biomass and hour was reached at the very beginning. Surprisingly, after a decrease in the acid/solvent switch, the glucose consumption increased again and accompanied the T3–T4 transition state with the highest number of regulated genes.

**Table 1:** Specific rate of glucose utilization between time-points chosen for RNA-seq analysis

Samples	Time interval (h)	Specific glucose consumption rate (g.g^-1a^.h^− 1^)
T1-T2	3.5–6.0	5.16
T2-T3	6.0–8.5	2.20
T3-T4	8.5–13.0	2.71
T4-T5	13.0–18.0	2.50
T5-T6	18.0–23.0	1.59

^a^Values were calculated for the concentration of viable cells

### Mapping statistics

**Fig. 2:**
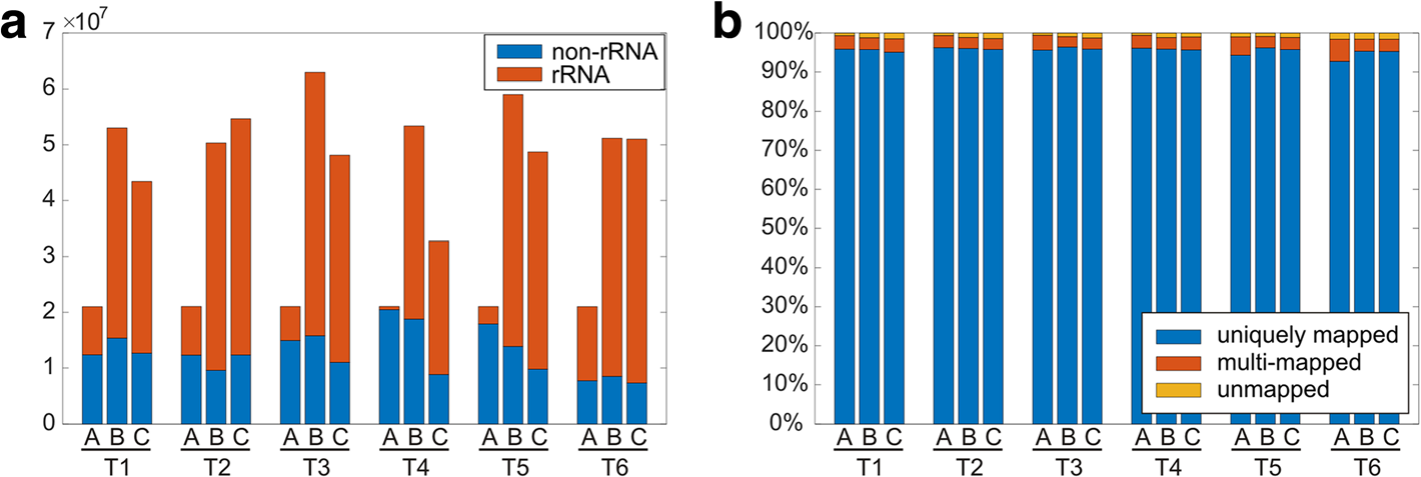
Quality of RNA-Seq reads. (**a**) The total number of reads in particular samples. The color of stacked bars distinguishes between non-rRNA and rRNA reads. (**b**) Mapping statistics of reads – percentages of uniquely mapped, multi-mapped, and unmapped non-rRNA reads

The whole dataset covered three series of six samples (six time-points), in which each series represented an independent biological replicate (A, B, and C). Although series A consisted of reads that were 50 bp long and series B and C consisted of reads that were 75 bp long, the whole series could be processed in the same way. The quality assessment after the first preprocessing steps (demultiplexing, quality trimming, and adapter trimming) confirmed an overall high-quality of sequences (average Phred score Q ≈ 35) and no adapter content. The only following sequence-filtering step was the removal of the remaining residual rRNA contamination, even after the rRNA depletion. The rRNA depletion was performed prior to the library construction and the non-captured rRNAs were apparent from the high GC content in some reads. The amount of non-rRNA reads ranged from 7.3 to 20.5 million (see Fig. 2a). Subsequently, we mapped the cleansed reads to the *C. beijerinckii* NRRL B-598 genome. Most reads mapped to the genome unambiguously, regardless of their different length in replicates A and B, C (see Fig. 2b). Nevertheless, in order to cover the expression of duplicated genes that were present in the *C. beijerinckii* NRRL B-598 genome, the reads mapping to multiple loci were also included in the gene expression analysis (see Table 2). However, the contribution of such reads was down-weighted in the expression analysis depending on the number of times they mapped to the genome, so the sum of the total number of reads stayed intact.

**Table 2:** Transcriptional activity of genes and pseudogenes

Sample	T1 (3.5 h)	T2 (6 h)	T3 (8.5 h)	T4 (13 h)	T5 (18 h)	T6 (23 h)	Total
No. of genes with RPKM>1^a^	5055 (4981)	5101 (5026)	5162 (5100)	5197 (5139)	5198 (5133)	5193 (5128)	5219 (5158)
No. of pseudogenes with RPKM>1^a^	188 (179)	186 (179)	190 (184)	196 (190)	195 (188)	194 (187)	197 (190)
Max. expression (RPKM)	4.0∙10^4^	3.4∙10^4^	3.4∙10^4^	3.4∙10^4^	3.4∙10^4^	4.0∙10^4^	4.0∙10^4^

^a^Values in brackets apply to uniquely mapped reads only

The reads mapping to more genomic objects were also weighted. Such a phenomenon is caused by overlapping genes. In the current RefSeq genome (NZ_CP011966.2), 285 out of the 5230 genes predicted by NCBI PGAP [14] overlapped by at least one codon and another 66 neighboring genes had no space between them. Although none of the 198 pseudogenes overlapped with another pseudogene, 18 pseudogenes overlapped with genes directly and another 73 pseudogenes were at a distance from genes that could be covered by a single read. These reasons caused single read mapping onto two genomic objects. At the same time, the transcriptome assembly contained fewer transcripts compared to the number of genomic elements with detectable transcription (precisely 4837 transcripts vs. 5418 genomic elements) because the overlapping and nearby genes, e.g. those in the same operon, were covered by a single transcript. Due to this fact, transcripts could not have been used to resolve overlapping genes. On the other hand, their mapping to the genome helped to confirm or disprove transcriptional activity of pseudogenes and prophages.

### Pseudogenes

Due to the high number of pseudogenes with detectable expression, we decided to further investigate their coverage by RNA-Seq reads. Only a single pseudogene remained completely silent when ambiguously mapping reads were used, while 184 pseudogenes had RPKM > 1 (Reads Per Kilobase per Milion mapped reads) in all six time-points. Using only uniquely mapped reads, eight pseudogenes remained completely silent and 178 were transcribed in every time-point. Although the number of transcribed pseudogenes remained almost the same across the six time-points, levels of their expression seemed to rise over time. While pseudogenes formed approximately 2.8% of *C. beijerinckii* NRRL B-598 genome, only 0.47% of all reads in T1 mapped to pseudogenes. However, this number continuously rose over the time according to the linear model %*mapped* = 0,1115 ∙ *time* - 0.0629 (with the regression value 0.9575), resulting in 2.83% of reads to be mapped onto pseudogenes in T6.

To further analyze the activity of pseudogenes, we decided to evaluate the coverage of pseudogenes through the use of transcripts assembled from all the reads in our dataset. The accuracy of mapping transcripts to the genome is higher thanks to their length (1057 bp on average). The results are summarized in Table 3.

**Table 3:** Coverage of pseudogenes by transcripts

	Not covered	Partly covered	Fully covered, overlapped transcripts	Fully covered, single transcript
Frameshifted	7	45	10	33
Missing start and/or stop	15	23	3	37
Internal stop	2	6	0	8
Combined issues	0	4	3	2
Total	24	78	16	80

There are 24 pseudogenes that were not covered by any transcript. These were probably completely silent (see Additional file 2). The second group consisted of 78 pseudogenes that were not covered in their whole length. In most cases, there were only short overlaps with transcripts of active genes neighboring these pseudogenes. In some cases, only part of a transcript was mapped to a pseudogene sequence, suggesting that these are silenced duplications of an active gene. Although genes in the third group were fully covered, this coverage consisted of two or more overlapping transcripts. Therefore, the transcription in both groups (partly covered and fully covered by overlapping transcripts) was highly questionable. On the contrary, pseudogenes within the fourth group were fully covered by unique transcripts. This group consisted of pseudogenes that were transcribed and active genes that were possibly misidentified as pseudogenes due to errors in the genome assembly. In comparison with their transcripts, 23 out of 80 pseudogenes (see Additional file 3) in this group were missing one nucleotide in homopolymers. This could have been caused by previous sequencing errors, as Roche 454 in combination with PacBio were used for the genome assembly. Nevertheless, insertion of these nucleotides was not detected in all reads mapping to these positions; the figure ranged from 60% to almost 100%.

### Transcription profiles and reproducibility

Only 11 genes were not transcribed at any of the six sampling points. Moreover, seven out of those 11 genes were related to 16S rRNA and these reads were filtered before mapping. Therefore, only four genes (X276_RS15615, X276_RS24570, X276_RS24585, X276_RS26445) demonstrated no transcripts. On the other hand, 5024 genes out of all 5219 transcribed genes (RPKM> 1) had detectable transcription at all time-points. Nevertheless, it is difficult to decide whether the expression of genes with low RPKM values has biological meaning, due to a high biological noise. Analysis using assembled transcripts is complicated, because most transcripts cover more than one gene and transcripts overlap. Transcription on a genome-wide scale (see Additional file 4) shows a novel pattern. While the transcriptional profiles from the first three time-points (T1, T2, and T3) correspond to the transcription of the *C. beijerinckii* NCIMB 8052 genome [5], the latter profiles do not.

**Fig. 3:**
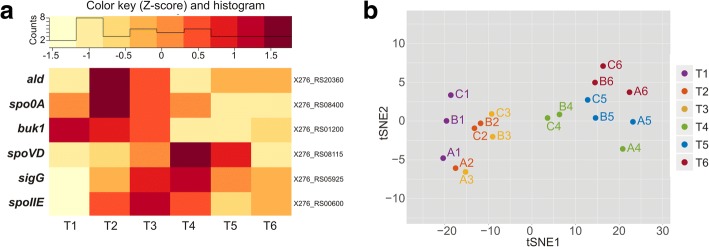
Analysis of the transcriptome reproducibility. (**a**) Transcription profiles of six selected genes visualized on the heatmap using a Z-score related to an average expression of each gene. (**b**) 2D representation of the normalized expression data after dimensionality reduction by t-SNE to compare the samples collected at the six time-points (T1–T6) coded by different colors. Each point represented a sample with a text label based on the biological replicate (A, B, and C) and the time-point from which it originated (T1–T6)

Reproducibility of the experiment was verified using three biological replicates and by checking the expression of six selected genes whose transcription profiles were observed during a previous study by Kolek et al. [11] (see Fig. 3a). The samples were visualized using the t-Distributed Stochastic Neighbor Embedding (t-SNE) [15] dimensionality reduction method on the normalized expression data. This final 2D representation showed that replicates (A, B, and C) were similar to each other at particular sampling times (T1–T6), while replicates sequenced using Illumina HiSeq (A) were slightly more distant to samples from Illumina NextSeq (B and C), see Fig. 3b. Overall, samples were divided into two clusters. While one cluster contained samples corresponding to the initial phase of fermentation (up to 8.5th hour), the other cluster consisted of samples from the later fermentation phase (from 13th up to 23rd hour).

### Differential expression

**Fig. 4:**
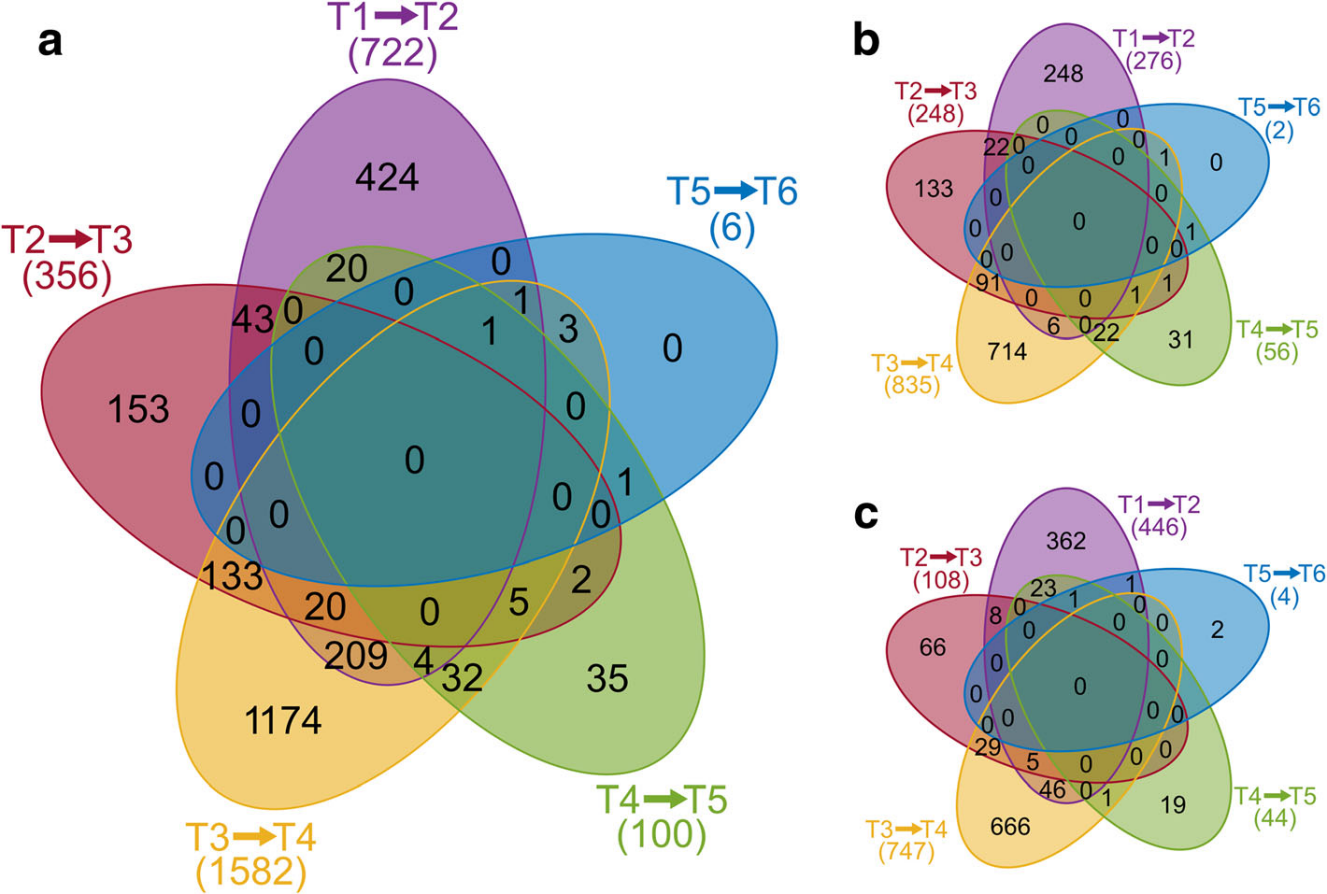
Differential expression analysis. Venn diagrams showing the number of (**a**) all-regulated, (**b**) up-regulated, and (**c**) down-regulated genes between adjacent time-points

We explored differential expression of all genes and pseudogenes with detectable transcription among adjacent time-points, in order to analyze changes in the transcription of particular genes over the whole fermentation process (see Fig. 4). In total, transcription of 2260 annotated genomic objects, forming more than 41.5% of all protein-coding elements, was regulated during the fermentation process when the criterion of adjusted *p*-value < 0.05 (Benjamini-Hochberg correction) was applied. While 474 genes were regulated more than once, only 31 of them were regulated more than three times. The single gene X276_RS14155 (PTS maltose transporter subunit IIBC) was regulated four times. The majority of differentially expressed genes were covered by at least 100 reads after the normalization of expression data (see Additional file 5). In total 3168 genes had no statistically significant regulations among adjacent time-points and formed potential housekeeping genes. The complete results of the differential expression analysis, including log2fold changes and adjusted *p*-values, are available in Additional file 6.

A major change was detected between the third and the fourth time-point when 1582 genes were regulated. While 835 out of these genes were up-regulated, 714 were up-regulated only between these two time-points (see Fig. 4b). Similarly, 666 out of the 747 down-regulated genes were down-regulated uniquely between T3 and T4 (see Fig. 4c). However, some of the uniquely up-regulated genes were down-regulated between another couple of time points and some of the uniquely down-regulated genes were up-regulated during another transition. Therefore, the total number of uniquely regulated genes between the T3 and T4 time-points was 1174. Every pair of adjacent time-points had uniquely regulated genes except for the last T5–T6 transition, when regulation of only six already regulated genes was detected. Nevertheless, previously up-regulated genes X276_RS05345 (hypothetical protein) and X276_RS24350 (butyrate kinase) were down-regulated between these later time-points. Both up-regulated genes during this transition, X276_RS08605 (tryptophan synthase subunit beta) and X276_RS18605 (DUF4179 domain-containing protein), also had detectable growth in transcription between previous time-points and were covered by more than 1000 and 2000 reads, respectively.

### Transcription of phage DNA

We searched the *C. beijerinckii* NRRL B-598 genome for phage sequences and found three prophages (see Table 4). While two of these regions were relatively short and phages were incomplete, the other phage was intact and consisted of 35 genes coding known phage proteins and six hypothetical protein-coding regions.

**Table 4:** Phage DNA within the *C. beijerinckii* NRRL B-598 genome

Region	Position	Length (bp)	Status	Total no. of proteins	No. of phage proteins
1	996,985..1006473	9488	incomplete	10	8
2	2,920,342..2960012	39,670	intact	41	35
3	4,005,361..4018720	13,357	incomplete	17	15

The expression within the first phage region corresponding to an incomplete phage was low (averaging RPKM = 47) with only two genes differentially expressed during T3–T4 change. Six genes were carried by a positive and four by a negative strand. Only four genes were fully covered by transcripts mapping to the region. The transcription within the third phage region covering the other incomplete phage was more active with average RPKM = 86, but none of the genes were differentially expressed during the fermentation. All genes were carried by a negative strand and 14 out of the 17 genes were covered by a single transcript, including one pseudogene (X276_RS17860) with a missing stop codon. The only region containing intact prophage consisted of 38 genes and three pseudogenes with a missing stop codon, carried by a positive strand. The whole region began with a pseudogene and had low transcription (averaging RPKM = 21). Although six genes had statistically significant differential expressions between T3 and T4, only short transcripts mapped to the region and only partly covered the genes. Thus, the phage remained silent.

## Discussion

The fermentation data presented in Fig. 1 comply with standard results usually achieved by using the same TYA cultivation medium [11, 12]. Deeper insight into the population is enabled by combination of double fluorescent staining and flow cytometry. Value of flow cytometry had already been confirmed for *C. acetobutylicum* [16, 17]. Cytometric data enabled the calculation of a specific rate of glucose consumption related to metabolically active cells in the population during different time periods of the cultivation, together with information about the overall culture condition.

The high proportion of reads that mapped to the genome in particular samples unambiguously, suggested a good quality of RNA-Seq data and successful alignment even for shorter 50 bp reads in replicates A. Although we presumed that utilization of longer 75 bp reads in replicates B and C could reach even higher percentage of unique mapping, the proportion remained similar (see Fig. 2b). Nevertheless, the number of genes with detectable transcription slightly differed when reads mapping to multiple loci were used. Although high sequencing depth and rRNA depletion brought a noise to RNA-Seq [18], in our case, this bias was caused by duplicated genes rather than being a sequencing issue [19]. To prevent omitting transcription of duplicated genes and pseudogenes, we decided to include multi-mapping reads into the analysis. The majority of reads mapped to the genome without any mismatches and support an overall high quality of the genome assembly. Nevertheless, 23 indels were detected in regions of frameshifted pseudogenes.

Although pseudogenes, in bacteria defined as ‘genes silenced by one or more deleterious mutations’ [20], could still be transcribed [21], their number in *C. beijerinckii* NRRL B-598 was rather high. For example, the reference sequence for the closely related strain *C. beijerinckii* NCIMB 8052 [13] (NC_009617.1) contained only 112 pseudogenes predicted by NCBI PGAP. While the number of pseudogenes with an incomplete coding region or those containing internal stop was comparable for both strains, the number of pseudogenes with frameshift was almost twice as high in *C. beijerinckii* NRRL B-598 genome. Although the high number of frameshifted genes could indicate an extraordinary number of frameshifted duplicates of genes, all 23 indels were detected in homopolymers. Therefore, such pseudogenes could also be misannotated genes due to pyrosequencing errors [22] that were not filtered out using PacBio RSII sequencing used for the complete genome assembly [9]. Nevertheless, 50 bp and 75 bp long reads were too short to distinguish between a frameshifted duplicate and an assembly error as no indels were present in 100% of reads mapping to ambiguous positions. Eventually, the activity of some pseudogenes was supported in differential expression analysis, by high log2foldchange, excessing a value of three.

The transcriptome of *C. beijerinckii* NRRL B-598 had never been studied before so no correlation to the older dataset could be carried out. However, the transcription of the six selected genes under the same cultivation conditions was monitored using qRT-PCR in study of *C. beijerinckii* NRRL B-598 and its mutant strain overexpressing sporulation initiation factor *spo0A* [11]. In the mentioned study by Kolek et al. [11], an increase in expression was observed in mid-cultivation for *spoIIE* and *sigG* and in the second part of cultivation for *spoVD*. This corresponded to the results of this study (see Fig. 3a). Moreover, the expression profiles of the remaining genes also showed the same pattern. Butyrate kinase (*buk,* X276_RS1200) transcription was maximal at the beginning of the cultivation, decreased in time, and rose slightly at the end of cultivation. The expression of *ald* and *spo0A* increased in the first third of cultivation and for *ald* also at the end of cultivation. Moreover, the reproducibility of the experiment was supported by utilization of three biological replicates and their high similarity in the sampling points visualized using tSNE in Fig. 3. The tSNE coordinates were obtained by comparing distances among samples in the original high-dimensional space, i.e. distances from the normalized expression profiles to the distances of the samples in the reduced space, i.e. the visualized points. The position of the samples in the 2D space was then optimized until the samples with similar expression profiles were placed close to each other and samples with very different expression profiles were at a further distance from each other. Two main clusters, distinguishing samples from the first and the second half of the experiment, were present. While the similarity of the replicates from the first cluster was supported mainly by the first coordinate tSNE1, the similarity in the other cluster was supported by the second coordinated tSNE2.

Wang et al. [13] observed similar clustering of RNA-Seq samples of *C. beijerinckii* NCIMB 8052, in which the first cluster was represented by samples from exponential and transition phases and the other by samples from a stationary phase. On the other hand, transcription profiles of *C. beijerinckii* NCIMB 8052 [5] and *C. beijerinckii* NRRL B-598 (see Additional file 4) on the genome-wide scale were different, especially in the later phase of cultivation. This could have been caused by structural reorganizations in the genomes of both strains or by differences in gene regulatory mechanisms. Due to the high similarity of both genomes (see Additional file 7), the latter seemed more relevant. The explanation for differences in transcription profiles of *C. beijerinckii* NRRL B-598 and *C. beijerinckii* NCIMB 8052 in the later phases could lie in the different phenotypic behavior of both strains at this stage. Although strain NCIMB 8052 ceased growing together with the start of solventogenesis [5, 13], strain NRRL B-598 continued growing until approximately half way through the solventogenic phase (see Fig. 1d). Another apparent difference was an increased number of mature spores formed by the NCIMB 8052 strain under similar cultivation conditions [12]. The genome of *C. beijerinckii* NRRL B-598 contained two housekeeping regions with stable high level of transcription activity that were not present in *C. beijerinckii* NCIMB 8052 genome. This high activity was caused by genes transcribing into cell wall binding proteins, in the first region by the gene X276_RS24890 with average RPKM 2.4∙10^4^, while in the second region by the gene X276_RS25120 with average RPKM 1.8∙10^4^. The most noticeable change in the transcription on the genome wide scale was captured between T3 and T4 time-points when the highest number of differentially expressed genes was detected. Increased activity was visible especially within the region spanning the position from 176,588 to 208,581 containing 45 genes whose average expression in RPKM rose from 1.9∙10^3^ to 3.0∙10^3^. Thirty-seven out of those genes code proteins belonged to the Clusters of Orthologous Groups of proteins (COG) functional group J associated with translation.

The massive change in the gene expression, which can be spotted in Fig. 4, was surprisingly not associated with the acidogenesis/solventogenesis switch that occurred earlier, mainly between the T2 and T3 time-points, neither with the sporulation initiation. Regarding the COG assignment of 45 abovementioned genes to group J (translation), it might be possible that at least a part of these genes corresponded with spore coat formation genes. Clostridial sporulation typically lasts 8–12 h and therefore the T4 time-point might have coincided with stage IV or V of a sporulation cycle in which formation of spore coat proteins occurred [23]. In addition to the coat proteins, a need for specific protein complexes involved in spore structures assemblies could be responsible for the increased protein formation demand.

Further transition between T4 and T5 could also show an entry to the irreversible phase of sporulation, in which two independent gene regulations were established in the mother cell and pre-spore and sporulation must be completed. Overall culture attenuation after T4 is apparent from both a decrease of specific glucose consumption (Table 1) and from cytometric data that confirmed the gradual increase in the proportion of inactive cells. An opposite phenomenon was observed between T3 and T4. An increase in the specific rate of glucose consumption, corresponding to highly regulated genes coding for COG functional group C (energy production and conversion) (see Additional file 8), was detected together with an apparently improved viability.

Even though the massive change between T3 and T4 was obvious, searching within COG categories (see Additional file 8) does not provide unambiguous clarification for this phenomenon. Mostly the same categories of regulated genes could be found between adjacent time-points within the first 13 h of cultivation with both down- and up-regulated representatives. After the 13th hour COG D and COG L related to cell cycle control and replication respectively were not differentially expressed which was fully consistent with the decrease in cell growth and declining culture viability supporting a hypothesis of the switch of a highly proliferating culture into a new strategy, securing genus preservation via ensuring a complete sporulation process. Simultaneously COG F for nucleotide metabolism transport are up-regulated within the first two compared time sets and down-regulated in the latter two. These findings were comparable to the transcriptional profile of *C. acetobutylicum* [24] unlike the category J which was in *C. acetobutylicum* down-regulated in the stationary phase. The same applied to the motility related genes (COG N) that were in our study more down-regulated even within the first measured interval and up-regulated in latter stages between T4 and T5. This might seem confusing as solventogenic clostridia are known to be motile within the exponential and acidogenic stage [25] and after the switch to solventogenesis, motility is generally lost. *C. beijerinckii* NRRL B-598 possessed such a change in motility as well but the first sample point T1 was already characterized by highly motile cells and therefore a decrease in related genes expression copied the phenotypic profile. On the other hand, an increase in the latter stages is probably the result of culture phenotype desynchronization when all the cell types are again present, including motile cells. The predominant upregulation of COG O (post translational modification, protein turnover, chaperone function) between later stages might relate to cell stress response to increasing solvent concentrations [26].

Furthermore, some cells within the whole population might have undergone a massive change in energy metabolism and solvent production, which is associated with the switch of different genes in the period of transition between T3 and T4 time-points. The solvent formation and acidogenesis/solventogenesis switch are usually explained as a stress response induced by accumulation of acids in the cultivation medium and pH decrease. Low pH could cause depletion of ATP pool in cells because of active transport of H^+^ ions across cell membrane. To prevent this event and to ensure population survival, some cells initiated sporulation, while other cells began converting acids into solvents. However, the whole population situation was no longer critical at time-point T4 and the lower concentration of acids in the medium might have induced another metabolic change, this time associated with the direct formation of butanol/acetone from glucose. As this pathway generated only a half of ATP in comparison with acidogenesis, its overall rate was probably higher. However, a significant advantage of the reduced risk of low pH outweighed this discomfort. Moreover, this hypothesis was supported by metabolites formations, glucose consumption, and pH profile (see Fig. 1a, c) and by an increase in specific glucose consumption. More than 20 years ago, Dürre et al. [27] envisaged for *C. acetobutylicum* that different genes are probably involved in early and late solventogenesis. Population heterogeneity reflected by FC and fluorescent staining (Fig. 1b) supports the hypothesis that not all cells in the population exhibit the same phenotype to cope with changing unfavorable living conditions. The population might rather choose the bet hedging strategy [28] to enable at least some cells from the population to survive.

Many bacterial genomes contain prophages or at least their remnants. Although they may represent large fraction of the strain-specific DNA sequences [29], the strain *C. beijerinckii* NRRL B-598 contained only three prophage regions while only one was complete. This could be the reason for a high genome sequence similarity with the strain *C. beijerinckii* NCIMB 8052 as the prophages are responsible for genome rearrangements and inversions [30]. Even though the complete prophage contained six differentially expressed genes between T3 and T4, their average transcription was very low suggesting false positive detection. Due to the absence of transcripts mapping to the prophage regions, all these three regions seemed to be silent. During industrial cultivations in the South Africa [31], there were several events mapped in which bacteriophages caused total collapse or reduction of solvents production due to lytic or lysogenic cycles, respectively. Therefore, the detected prophages deserve further experimental investigation.

## Conclusions

Although the strain *C. beijerinckii* NRRL B-598 is a promising butanol producer, we lack a precise description of mechanisms within its fermentation metabolism, which prevent us from further modifications of the strain for industrial applications. Moreover, these mechanisms seems to be unique and different from other clostridia, including a closely related strain *C. beijerinckii* NCIMB 8052. In this study, we provided a complex analysis of its fermentation profile using HLPC, FC, and RNA-Seq technologies. Six time-points were selected to study its transcription profile, while the whole experiment was repeated in order to get three biological replicates (A, B, and C) for each time-point. This allowed us to verify the reproducibility of the experiment and to gather the RNA-Seq dataset with the currently highest dynamic range available among solventogenic clostridia. We analyzed the latest RefSeq annotation of the genome and confirmed its high accuracy. Nevertheless, through the analysis of single nucleotide variants, several putative missing nucleotides were found within the regions of frameshifted pseudogenes. Transcription regulations identified by differential expression analysis of adjacent time-points showed the greatest changes between T3 and T4 time-points. Surprisingly, this change was not directly connected to the acidogenic/solventogenic change, nor the sporulation initiation but rather to a massive change in the energy metabolism and solvent production in a part of cell population as we discuss based on auxiliary HLPC and FC data.

Furthermore, we discovered three prophage regions within the genome, which demonstrated low or no transcription activity. Nevertheless, these regions are important for further experimental investigation. The experimental design and the gathered data proved good reproducibility, therefore, repeating the experiment under different conditions will also allow us to explore gene regulatory mechanisms and signaling pathways within the strain.

## Methods

### Bacterial culture and fermentation experiment

The strain *C. beijerinckii* NRRL B-598 was maintained in a form of spore suspension. TYA broth, prepared according to Kolek et al. (2017) [11], containing: 50 g/l glucose, 6 g/l tryptone (Sigma Aldrich), 2 g/l yeast extract (Merck), 3 g/l ammonium acetate, 0.5 g/l KH_2_PO_4_, 0.3 g/l MgSO_4_∙7H_2_O, and 0.01 g/l FeSO_4_, was used for the fermentation experiment. Multiforce 1 l bioreactors (Infors HT) with 630 ml TYA broth and agitation at 200 rpm were used for batch cultivation of the strain at 37 °C. Oxygen was removed from bioreactors by bubbling with N_2_ prior to fermentation. pH was adjusted to 6.3 by 10% NaOH and all bioreactors were inoculated with 70 ml of inoculum that was cultured previously in an anaerobic chamber overnight (Concept 400; Ruskinn Technology) under an anaerobic atmosphere (90% N_2_, 10% H_2_). The whole experiment was repeated during different weeks to obtain three biological replicates.

Samples were taken at specific times and processed for cell concentration determination, HPLC analysis, microscopy, flow cytometry, and RNA isolation. Samples for RNA isolation were taken at 3.5, 6, 8.5, 13, 18, and 23 h of cultivation.

### Culture growth and HPLC analysis

Cell concentration was determined by the optical density (OD) measurement at 600 nm with Spectrophotometer (Varian Cary 50 UV-VIS spectrophotometer, Varian) against TYA broth. For calculations of a specific glucose consumption rate, dry weight of biomass (CDW) was used. CDW was determined after drying biomass until constant weight at 105 °C. The equation was following:

$$ {q}_p=\frac{c_{i+1}-{c}_i}{\overline{CDW_{i;i+1}}\ast \overline{X_{i;i+1}}\ast \left({t}_{i+1}-{t}_i\right)} $$

where *q*_*p*_ is a specific substrate consumption rate related to a number of viable cells (g.g^− 1^.h^− 1^), *c* is concentration of glucose (g/L), *CDW* is cell dry weight (g/L), *x* is a proportion of viable cells in population and *t* is time (h). Symbols *i* and *i + 1* indicate two adjacent sampling time points.

Concentrations of glucose and fermentation products (lactic acid, acetic acid, butyric acid, ethanol, acetone, and butanol) were measured by HPLC with refractive index detection (Agilent Series 1200 HPLC; Agilent) in microfiltered samples of culture broths. An IEX H+ polymer column (Watrex) was used for the separation. Conditions of analysis were as follows: isocratic elution, 5 mM H_2_SO_4_ as a mobile phase with flow rate of 0.5 ml min^− 1^, column temperature 60 °C, injection sample volume 20 μl. The chromatograms were processed by ChemStation for LC systems software using a set of standard samples with known concentrations to elaborate calibration curves.

### Microscopy, fluorescent staining, and flow cytometry

Phase contrast microscopy (Olympus BX51; Olympus) with × 400 and × 1000 magnifications was used to determine the morphological status of cells. Population viability and heterogeneity was evaluated using flow cytometry (BD Accuri C6) in combination with fluorescent staining. A combination of propidium iodide PI (Sigma Aldrich) and carboxyfluorescein diacetate CFDA (Sigma Aldrich) was employed for the differentiation of active and damaged cells and detection of spores according to Kolek et al. (2016) [12].

### RNA isolation and sequencing

Cell samples for isolation of total RNA were collected from 3 ml of culture broth (OD_600_ 0.9–1.0) by centrifugation at 10000 rpm for two minutes, washed with RNase free water and cell pellets were immediately stored at − 70 °C. RNA from the cell pellet was isolated using High Pure RNA Isolation Kit (Roche). Isolated total RNA was stored frozen at − 70 °C. The total RNA concentration was determined on DS-11 FX+ Spectrophotometer (DeNovix). Quality and integrity of the samples were assessed using the Agilent RNA 6000 Nano Kit (Agilent) with the Agilent 2100 Bioanalyzer (Agilent). RNA integrity number was measured using 2100 Bioanalyzer Expert software.

Frozen total RNA samples were thawed on ice and an aliquot of each sample containing 10 μg of RNA was taken for 16S and 23S ribosomal RNAs removal using The MICROB*Express*™ Bacterial mRNA Enrichment Kit (Ambion). Efficiency of ribosomal RNA depletion and concentration of RNA samples were checked on the Agilent 2100 Bioanalyzer (Agilent) with the Agilent RNA 6000 Nano Kit (Agilent). Library construction and sequencing of samples from the first replicate on Illumina HiSeq 4000, single-end, 50 bp, was performed by BGI Europe A/S (Copenhagen, Denmark). Library construction and sequencing of samples from two remaining replicates were performed by CEITEC Genomics core facility (Brno, Czechia) on Illumina NextSeq, single-end, 75 bp.

### Bioinformatics analysis

The quality assessment after steps of the RNA-Seq reads processing was done using FastQC in combination with MultiQC to summarize the reports across all samples [32]. Reads representing 16S and 23S rRNA regions were filtered out using SortMeRNA [33] with SILVA database of known bacterial 16S and 23S rRNA genes [34] to simplify the following mapping of reads. Clean reads were mapped to the reference genome of *C. beijerinckii* NRRL B-598 (NZ_CP011966.2) using STAR [35]. Resulting SAM (Sequence Read Alignment/Map) files were indexed and transformed into more compact BAM (Binary Read Alignment/Map) format using SAMtools [36].

Transcripts were assembled de novo from a whole dataset of 18 samples using Trinity v2.4.0 [37]. Transcripts were mapped to *C. beijerinckii* NRRL B-598 reference genome (NZ_CP011966.2) with BLAST+ v2.7.1 [38]. Mapped reads and transcripts were visualized as a graph of sequence read coverage across the genome and further explored in Integrative Genomics Viewer (IGV) v 2.4.3 [39] to capture variable regions, including identification of putative missing nucleotides in pseudogene region in the current genome assembly. On the other hand, genome-wide coverage plots were reconstructed with SAMtools using sorted reads and visualized as circular representations of genome with DNAplotter [40] integrated in Artemis [41]. Dotplot for visual comparison of *C. beijerinckii* NRRL B-598 and *C. beijerinckii* NCIMB 8052 genomes was produced in YASS genomic similarity search tool [42]. Phage regions in the *C. beijerinckii* NRRL B-598 genome were predicted with PHASTER [43] and PhiSpy [44]. In PhiSpy both available clostridial references (*C. perfringens* and *C. tetani*) were used.

A count table was reconstructed using the R/Bioconductor featureCounts function included in the Rsubread package [45] and RPKM were computed using the R/Bioconductor edgeR package [46]. Differential analysis was performed on a raw count table with R/Bioconductor DESeq2 package [47]. Data was normalized using a built-in DESeq2 function. This normalization used negative binomial distribution and handles both differences in library sizes and differences in library composition. DESeq2 identified genes that were differentially expressed in a time-dependent manner. Dimensionality reduction and visualization of normalized samples was produced with R Rtsne package using Barnes-Hut t-SNE implementation [48] in combination with ggplot2 R package [49]. Venn diagrams and heatmaps representing transcription of selected genes using Z score were generated with R packages VennDiagram [50] and gplots, respectively. Time series and bar plots were generated with Matlab 2017b.

## Additional files

Additional file 1: Snapshots from microscopic observation during cultivation. (PDF 628 kb)

Additional file 2: Silent pseudogenes. (PDF 195 kb)

Additional file 3: Putative active genes misidentified as pseudogenes due to assembly errors. (PDF 210 kb)

Additional file 4: Circular plots showing average coverage of the genome by RNA-Seq reads in all six time points. The outermost and the second outermost circles represent positions of genes on the forward (red) and reverse (blue) strands respectively. The third circle (green) stands for pseudogenes. The yellow peak and shading area represents transcription greater than the average and violet lower than average. Floating window of 10,000 bp with step of 200 bp was used to render the shading area. (PDF 701 kb)

Additional file 5: Differential analysis of adjacent time points using MA plots. MA plots showing statistically differentially expressed genes in color. Color coding respect the color coding used in Venn diagrams in Fig. 4. (PDF 315 kb)

Additional file 6: Differential expression analysis. Complete results of differential expression analysis using DESeq2. (XLSX 2287 kb)

Additional file 7: Dotplot of *C. beijerinckii* NRRL B-598 and *C. beijerinckii* NCIMB 8052 genome. Dotplots showing that no major rearrangement between the two strains are present. (PDF 315 kb)

Additional file 8: COG functional categories of differential expressed genes. Barplots showing the number of COG categories associated with differentially expressed genes between adjacent time points. (PDF 226 kb)
