# Supplementary material for: Transcription profiling of butanol producer Clostridium beijerinckii NRRL B-598 using RNA-Seq
Source: BMC Genomics. 2018 May 30;19:415. doi: 10.1186/s12864-018-4805-8 (PMC5975590; doi:10.1186/s12864-018-4805-8)
Supplement: Supplementary file 1 — Snapshots from microscopic observation during cultivation. (PDF 628 kb) [file 12864_2018_4805_MOESM1_ESM.pdf]

Additional file 1: Snapshots from microscopic observation during cultivation

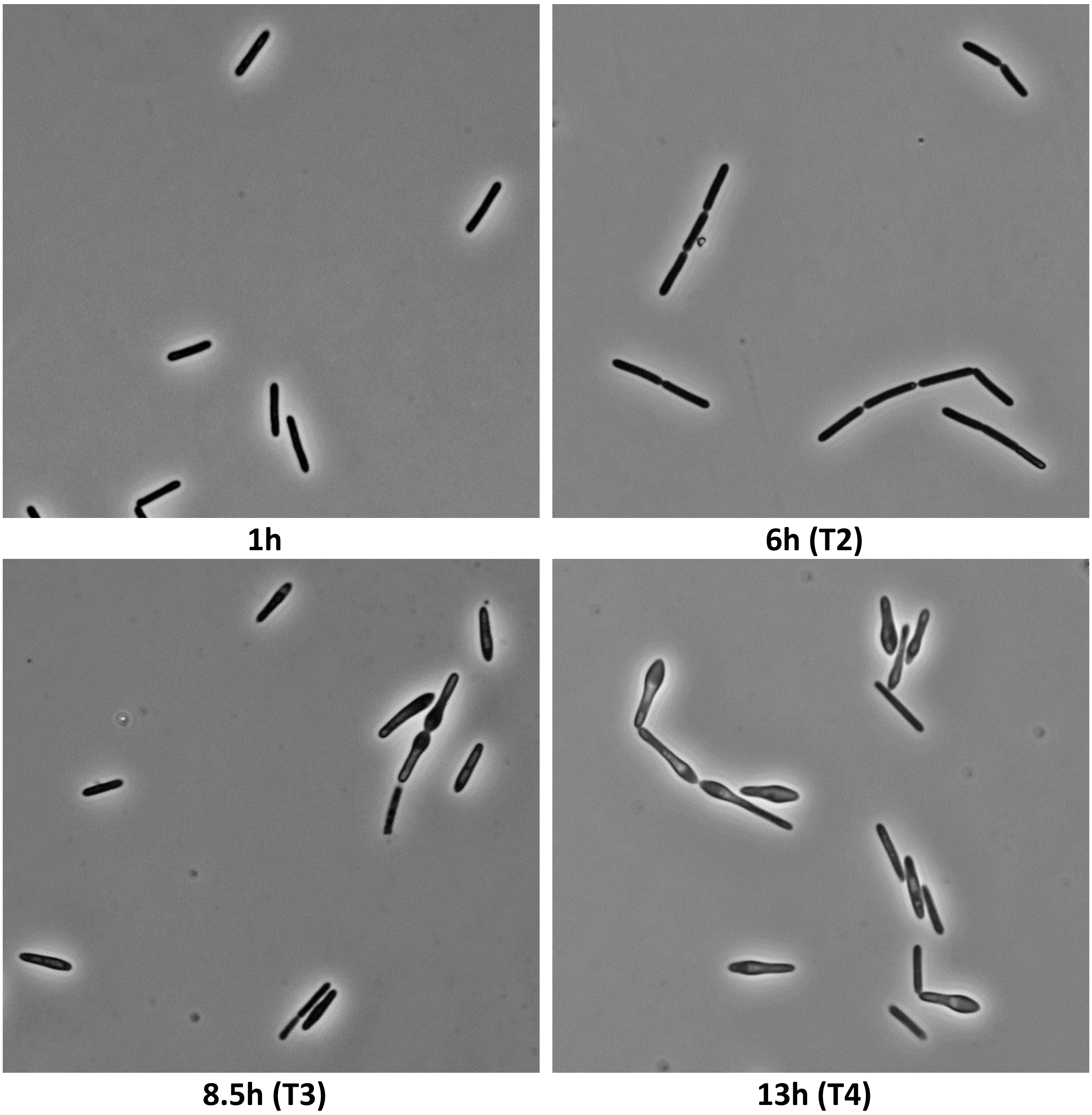

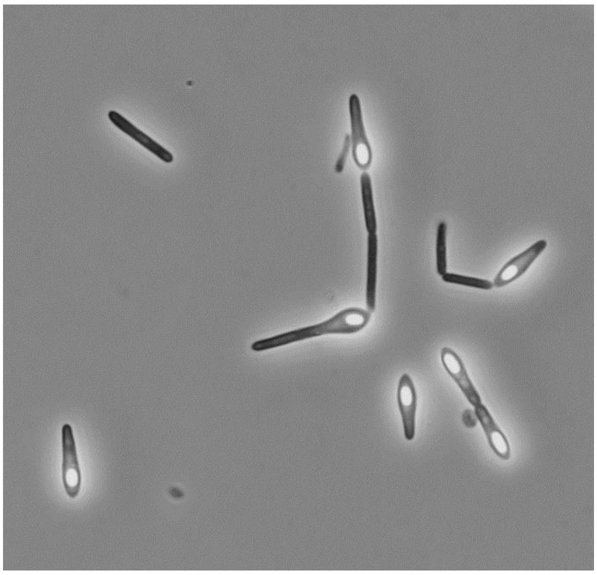

18h (T5)

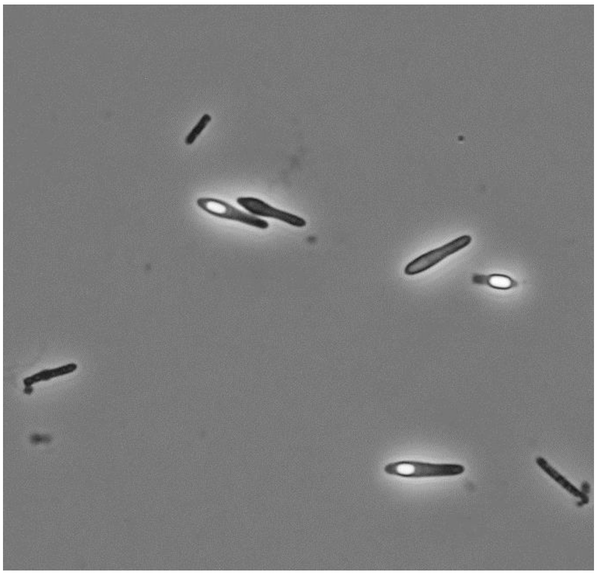

23h (T6)

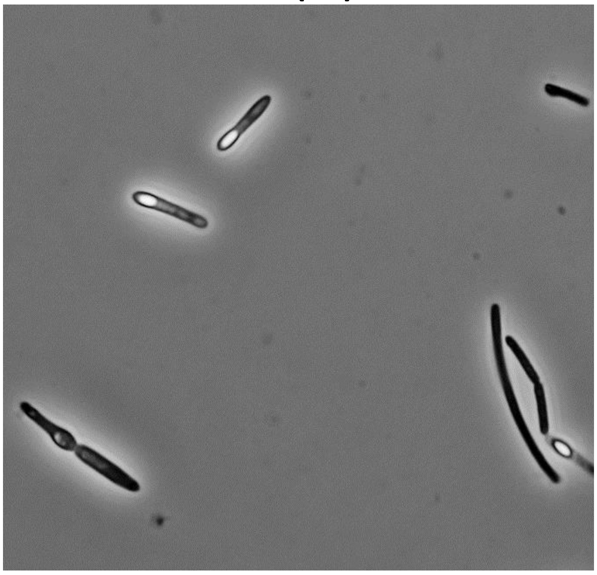

28h

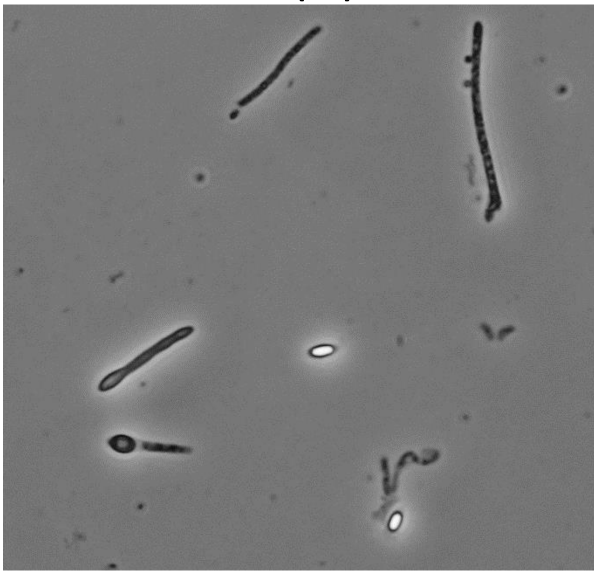

33h

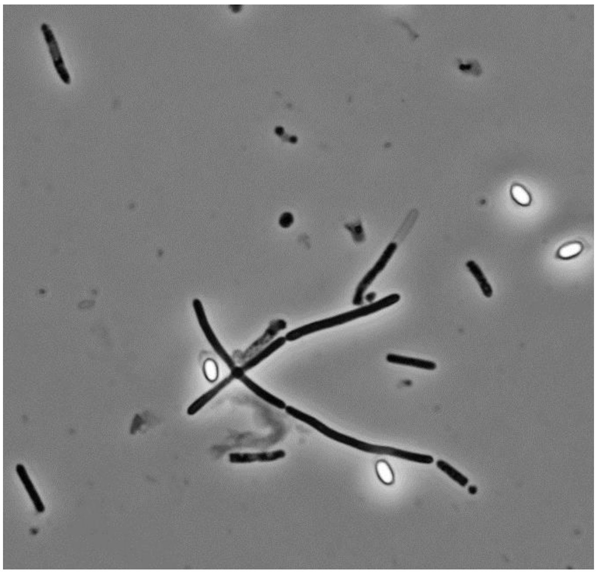

48h
