# Supplementary material for: Transcription profiling of butanol producer Clostridium beijerinckii NRRL B-598 using RNA-Seq
Source: BMC Genomics. 2018 May 30;19:415. doi: 10.1186/s12864-018-4805-8 (PMC5975590; doi:10.1186/s12864-018-4805-8)
Supplement: Supplementary file 2 — Silent pseudogenes. (PDF 195 kb) [file 12864_2018_4805_MOESM2_ESM.pdf]

Additional file 2: Silent pseudogenes

Locus tag	Length	Position	Reason	Description
X276_RS00260	255	57447..57701	missing start	two-component sensor histidine kinase
X276_RS07565	582	1689595..1690176	missing start	dioxygenase
X276_RS09090	90	1997165..1997254	missing start	transposase
X276_RS10345	345	2290821..2291165	missing start	fructose-6-phosphate aldolase
X276_RS10405	126	2307301..2307426	missing start	ATPase
X276_RS11265	297	2501800..2502096	frameshifted	hypothetical protein
X276_RS11380	252	2530485..2530736	missing start	NimC/NimA family protein
X276_RS11630	651	2572388..2573038	internal stop	polysaccharide deacetylase
X276_RS11650	428	2575278..2575705	frameshifted	hypothetical protein
X276_RS12805	1274	2847476..2848749	frameshifted	PTS galactitol transporter subunit IIC
X276_RS14035	444	3118166..3118609	missing start	4Fe-4S ferredoxin
X276_RS14730	459	3297305..3297763	missing start	hypothetical protein
X276_RS14985	379	3362166..3362544	missing start	3-beta hydroxysteroid dehydrogenase
X276_RS15030	581	3373053..3373633	frameshifted	hypothetical protein
X276_RS15335	485	3445278..3445762	frameshifted	hypothetical protein
X276_RS15510	309	3480030..3480338	missing start	40-residue YVTN family beta-propeller
X276_RS15520	363	3482406..3482768	missing start	alkyl hydroperoxide reductase
X276_RS15650	102	3505764..3505865	missing start	hypothetical protein
X276_RS17205	396	3860781..3861176	internal stop	hypothetical protein
X276_RS18855	296	4191322..4191617	frameshifted	hypothetical protein
X276_RS20640	759	4603594..4604352	missing stop	histidine kinase
X276_RS20720	364	4620598..4620961	missing stop	hypothetical protein
X276_RS21535	1012	4798160..4799171	frameshifted	DNA-binding response regulator
X276_RS26610	285	6080564..6080848	missing stop	hypothetical protein
