# Supplementary material for: Transcription profiling of butanol producer Clostridium beijerinckii NRRL B-598 using RNA-Seq
Source: BMC Genomics. 2018 May 30;19:415. doi: 10.1186/s12864-018-4805-8 (PMC5975590; doi:10.1186/s12864-018-4805-8)
Supplement: Supplementary file 3 — Putative active genes misidentified as pseudogenes due to assembly errors. (PDF 210 kb) [file 12864_2018_4805_MOESM3_ESM.pdf]

Additional file 3: Putative active genes misidentified as pseudogenes due to assembly errors

Locus tag	Length	Position	Reason	Description	RPKM					
					3.5h	6h	8.5h	13h	18h	23h
X276_RS05310	1250	1181908..1183157	frameshifted	phosphoribosylamine--glycine ligase	38	59	202	64	48	53
X276_RS06945	722	1541349..1542070	frameshifted	1-(5-phosphoribosyl)-5-[(5-phosphoribosylamino)methylideneamino]imidazole-4-carboxamide isomerase	100	82	74	83	73	64
X276_RS07745	797	1717089..1717885	frameshifted	TIGR00266 family protein	21	29	32	39	37	31
X276_RS08460	2219	1861491..1863709	frameshifted	dolichyl-phosphate-mannose--protein mannosyltransferase	29	50	33	18	20	38
X276_RS08755	2645	1929674..1932318	frameshifted	valine--tRNA ligase	96	94	68	60	46	45
X276_RS10200	238	2254697..2254934	comb. issues	hypothetical protein	385	403	325	149	125	121
X276_RS12380	1025	2740860..2741884	frameshifted	cell wall-binding protein	90	116	102	215	176	109
X276_RS12465	1091	2766820..2767910	frameshifted	phosphoribosylpyrophosphate synthetase	135	101	97	108	88	74
X276_RS13545	1058	2999949..3001006	frameshifted	glycosyl transferase family 1	23	15	14	278	499	423
X276_RS13575	1625	3011981..3013605	frameshifted	glycosyl transferase	25	21	28	242	377	277
X276_RS13580	935	3013800..3014734	frameshifted	glycosyl transferase	67	40	151	395	351	145
X276_RS14990	1109	3362648..3363756	frameshifted	Fe-S oxidoreductase	231	295	545	688	490	306
X276_RS15385	473	3457842..3458314	frameshifted	spore coat protein	46	19	62	709	514	216
X276_RS15660	1169	3507034..3508202	frameshifted	sugar kinase	78	72	35	25	30	47
X276_RS16200	1256	3636812..3638067	frameshifted	amino acid permease-associated protein	10	12	14	25	18	19
X276_RS18130	722	4052581..4053302	frameshifted	hypothetical protein	35	32	24	20	15	16
X276_RS20935	1046	4662125..4663170	frameshifted	galactose ABC transporter substrate-binding protein	37	48	55	89	78	57
X276_RS21490	1685	4788285..4789969	frameshifted	2-oxoacid:ferredoxin oxidoreductase subunit alpha	44	26	112	436	354	166
X276_RS21750	1715	4846544..4848258	frameshifted	dihydroxy-acid dehydratase	13	40	68	79	122	199
X276_RS22255	1043	4975478..4976520	frameshifted	sulfate transporter subunit	198	135	87	90	146	326
X276_RS23140	1079	5190079..5191157	frameshifted	undecaprenyldiphospho-muramoylpentapeptide beta-N-acetylglucosaminyltransferase	32	61	61	57	64	47
X276_RS23155	2912	5194118..5197029	frameshifted	DNA topoisomerase IV	72	84	76	54	41	46
X276_RS23865	1706	5377008..5378713	frameshifted	hybrid sensor histidine kinase/response	36	38	27	18	21	26
