# Supplementary material for: Transcription profiling of butanol producer Clostridium beijerinckii NRRL B-598 using RNA-Seq
Source: BMC Genomics. 2018 May 30;19:415. doi: 10.1186/s12864-018-4805-8 (PMC5975590; doi:10.1186/s12864-018-4805-8)
Supplement: Supplementary file 4 — Circular plots showing average coverage of the genome by RNA-Seq reads in all six time points. The outermost and the second outermost circles represent positions of genes on the forward (red) and reverse (blue) strands respectively. The third circle (green) stands for pseudogenes. The yellow peak and shading area represents transcription greater than the average and violet lower than average. Floating window of 10,000 bp with step of 200 bp was used to render the shading area. (PDF 701 kb) [file 12864_2018_4805_MOESM4_ESM.pdf]

# Additional file 4: Circular plots showing average coverage of the genome by RNA-Seq reads in all six time points

The outermost and the second outermost circles represent positions of genes on the forward (red) and reverse (blue) strands respectively. The third circle (green) stands for pseudogenes. The yellow peak and shading area represents transcription greater than the average and violet lower than average. Floating window of 10,000 bp with step of 200 bp was used to render the shading area.

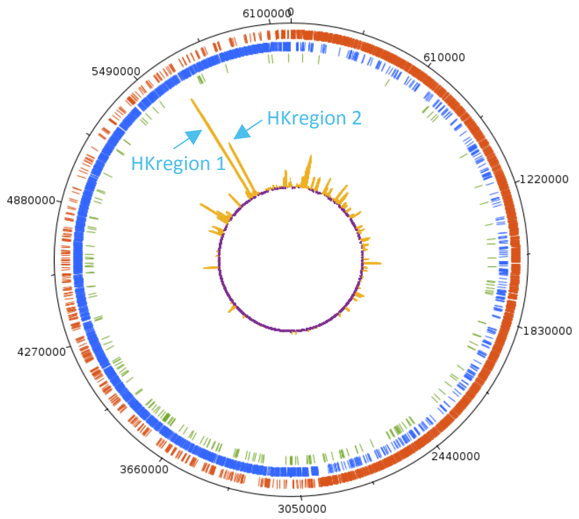


**T1 (3.5h)**

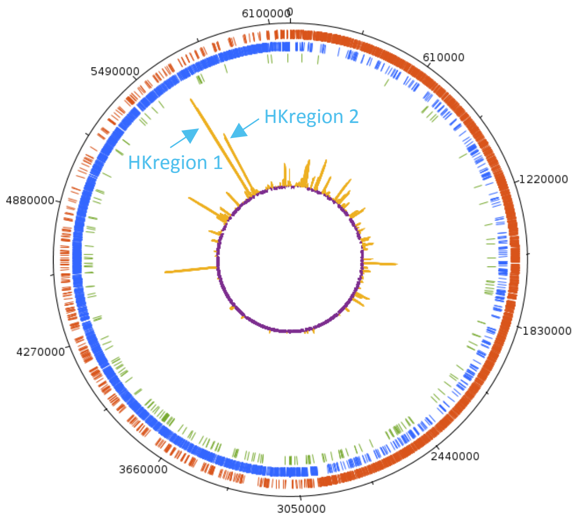


**T2 (6h)**

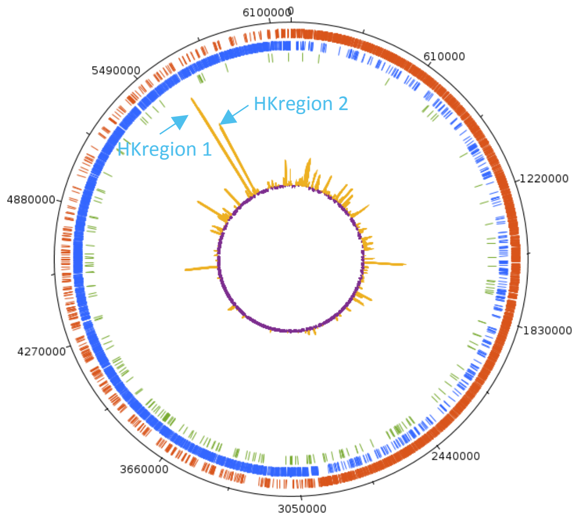


**T3 (8.5h)**

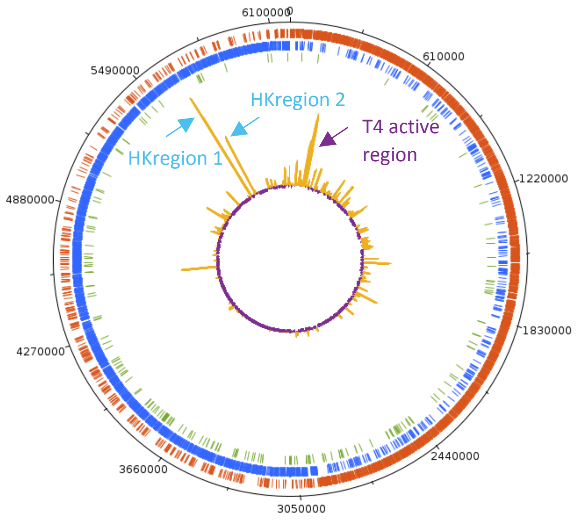


**T4 (13h)**

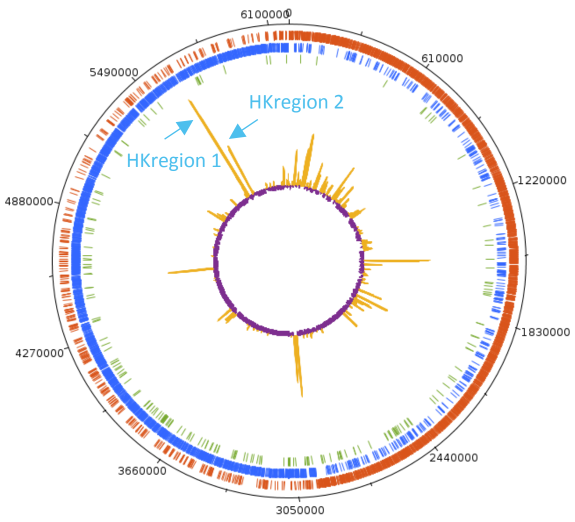
0
6100000
610000
5490000
1220000
4880000
1830000
4270000
2440000
3660000
3050000
HKregion 1
HKregion 2

**T5 (18h)**

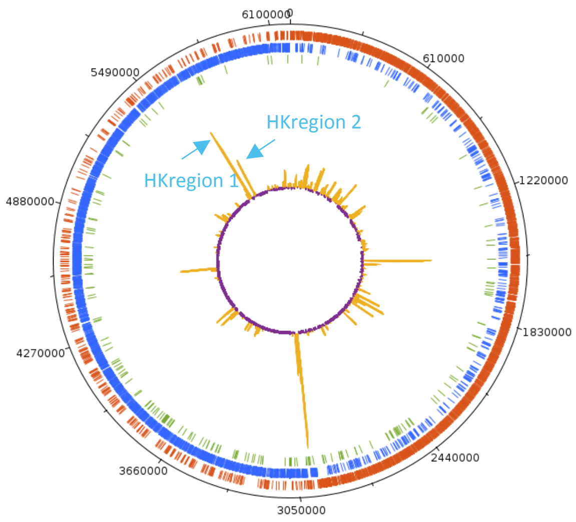
0
6100000
610000
5490000
1220000
4880000
1830000
4270000
2440000
3660000
3050000
HKregion 1
HKregion 2

**T6 (23h)**
