# Supplementary material for: Transcription profiling of butanol producer Clostridium beijerinckii NRRL B-598 using RNA-Seq
Source: BMC Genomics. 2018 May 30;19:415. doi: 10.1186/s12864-018-4805-8 (PMC5975590; doi:10.1186/s12864-018-4805-8)
Supplement: Supplementary file 5 — Differential analysis of adjacent time points using MA plots. MA plots showing statistically differentially expressed genes in color. Color coding respect the color coding used in Venn diagrams in Fig. 4. (PDF 315 kb) [file 12864_2018_4805_MOESM5_ESM.pdf]

Additional file 5: Differential analysis of adjacent time points using MA plots

MA plots showing statistically differentially expressed genes in color. Color coding respect the color coding used in Venn diagrams in Figure 4.

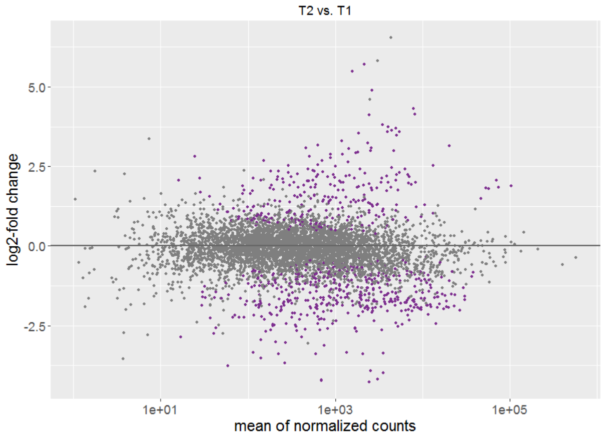

T1→T2

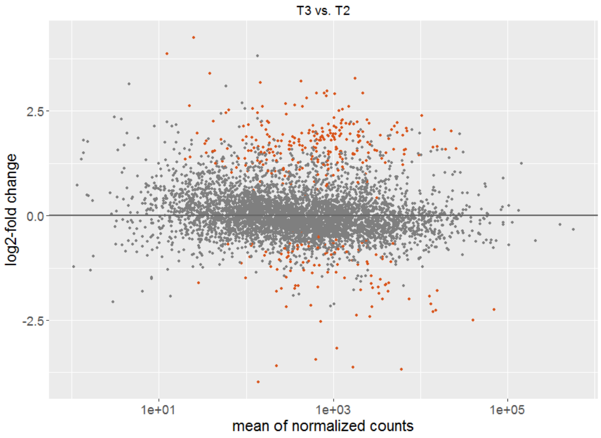

T2→T3

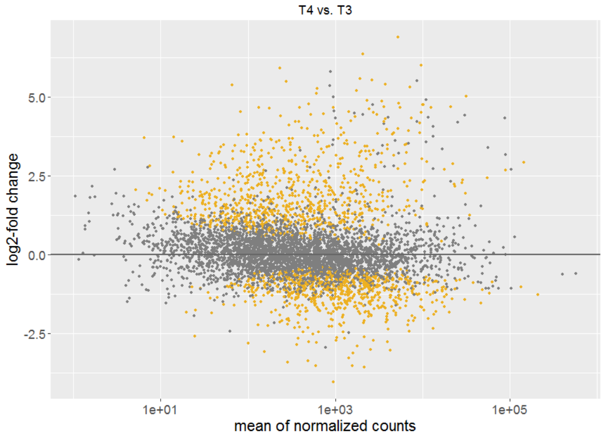

T3→T4

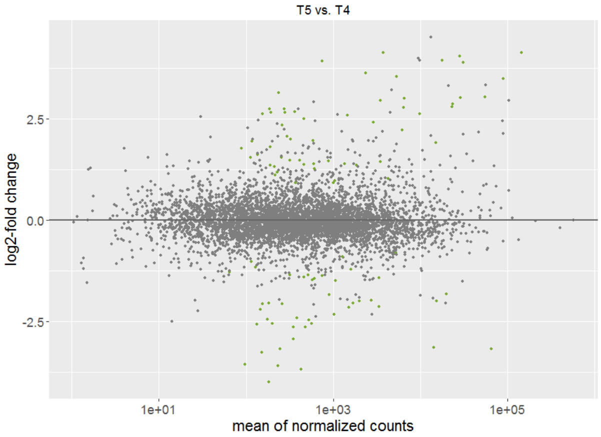

T4→T5

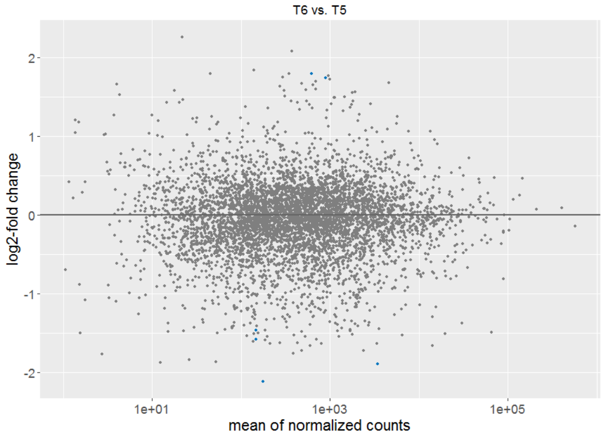

T5→T6
