# Supplementary material for: Transcription profiling of butanol producer Clostridium beijerinckii NRRL B-598 using RNA-Seq
Source: BMC Genomics. 2018 May 30;19:415. doi: 10.1186/s12864-018-4805-8 (PMC5975590; doi:10.1186/s12864-018-4805-8)

Additional file 7: Dotplot of *C. beijerinckii* NRRL B-598 and *C. beijerinckii* NCIMB 8052 genome

Doplots showing that no major rearrangement between the two strains are present.

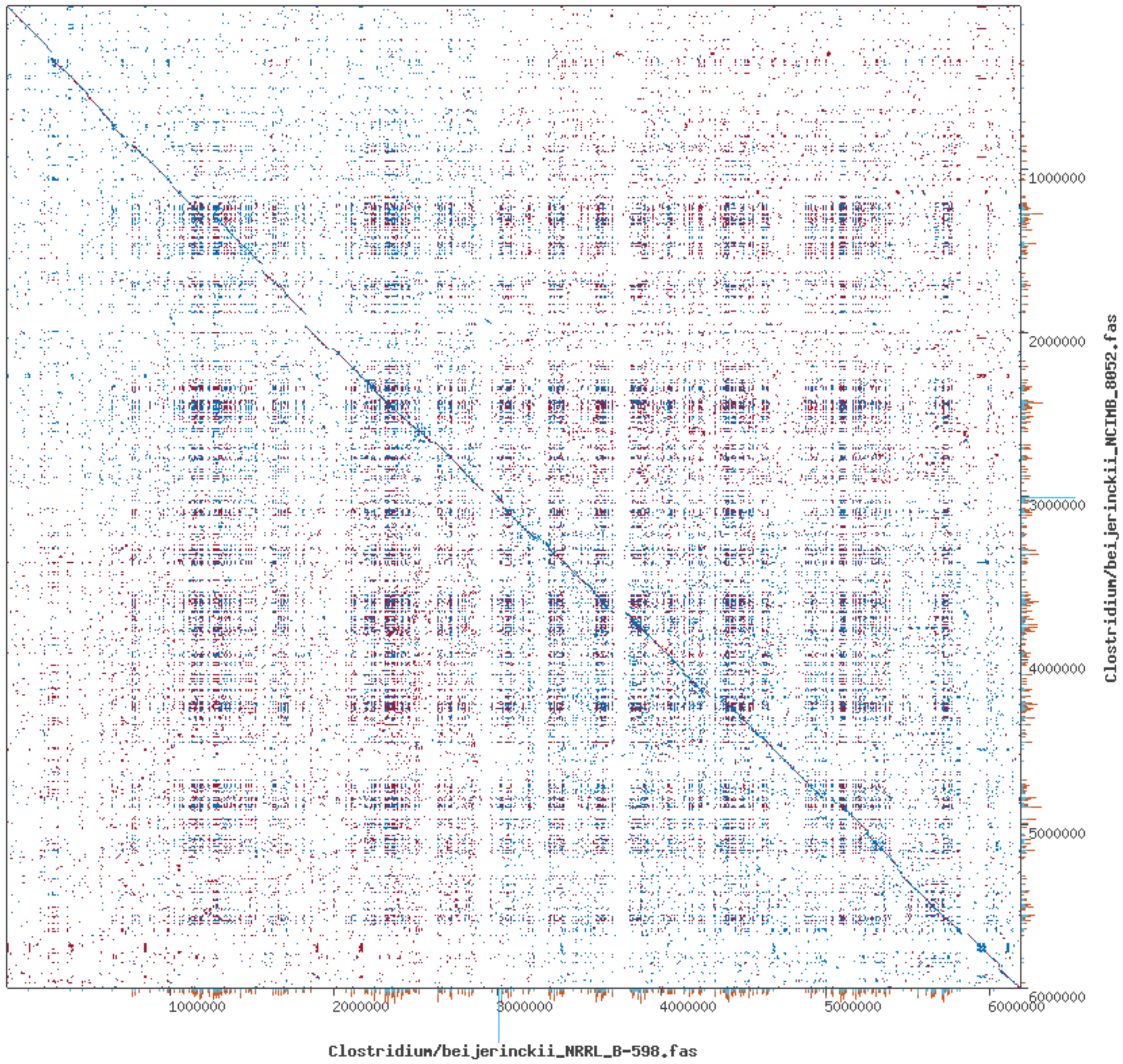

Supplement: Supplementary file 7 — Dotplot of C. beijerinckii NRRL B-598 and C. beijerinckii NCIMB 8052 genome. Dotplots showing that no major rearrangement between the two strains are present. (PDF 315 kb) [file 12864_2018_4805_MOESM7_ESM.pdf]
