# Supplementary material for: Transcription profiling of butanol producer Clostridium beijerinckii NRRL B-598 using RNA-Seq
Source: BMC Genomics. 2018 May 30;19:415. doi: 10.1186/s12864-018-4805-8 (PMC5975590; doi:10.1186/s12864-018-4805-8)
Supplement: Supplementary file 8 — COG functional categories of differential expressed genes. Barplots showing the number of COG categories associated with differentially expressed genes between adjacent time points. (PDF 226 kb) [file 12864_2018_4805_MOESM8_ESM.pdf]

## Additional file 8: COG functional categories of differential expressed genes

Barplots showing the number of COG categories associated with differentially expressed genes between adjacent time points.

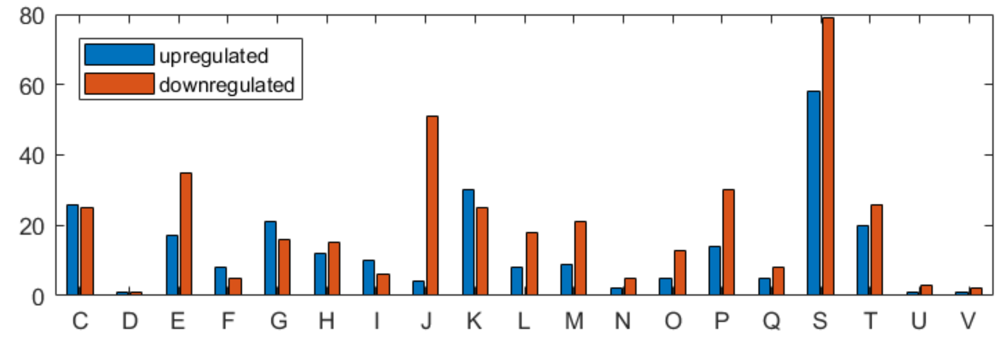


**T1→T2**

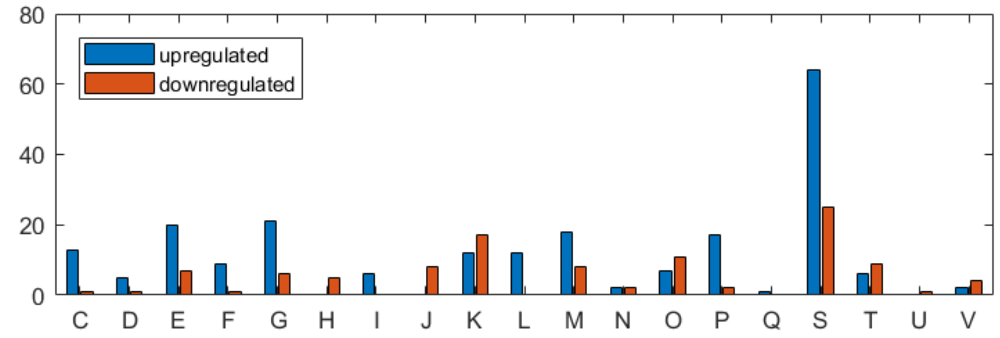


**T2→T3**

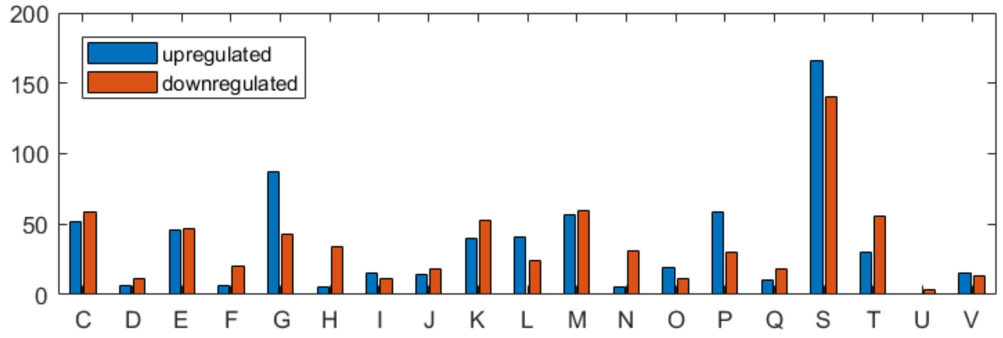


**T3→T4**

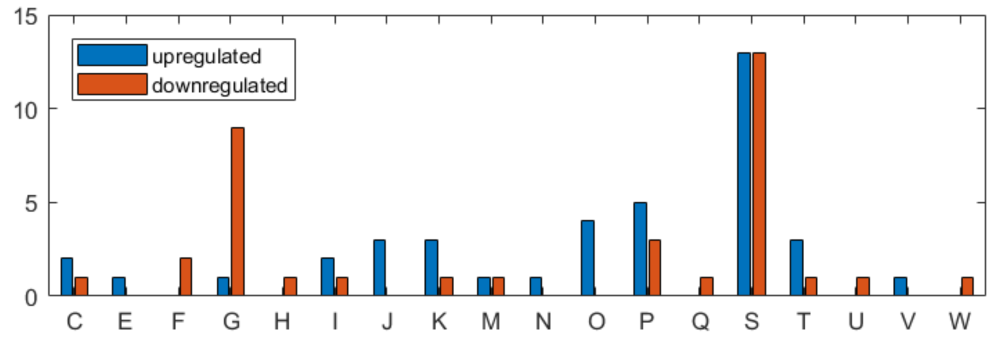
15
10
5
0
upregulated
downregulated
C
E
F
G
H
I
J
K
M
N
O
P
Q
S
T
U
V
W

**T4→T5**

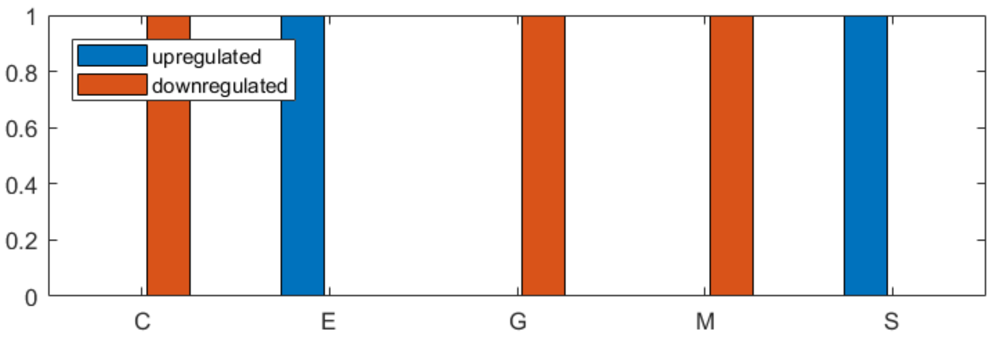
1
0.8
0.6
0.4
0.2
0
upregulated
downregulated
C
E
G
M
S

**T5→T6**
